# Differences in the localization of AQP1 and expression patterns of AQP isoforms in rat and mouse sciatic nerve and changes in rat AQPs expression after nerve crush injury

**DOI:** 10.1016/j.ibneur.2021.12.004

**Published:** 2021-12-24

**Authors:** Edith Segura-Anaya, Alejandro Martínez-Gómez, Myrna A.R. Dent

**Affiliations:** Laboratorio de Neurociencias, Facultad de Medicina, Universidad Autónoma del Estado de México, Paseo Tollocan y Jesús Carranza, Toluca, Edo. de México CP 50180, México

**Keywords:** Peripheral nervous system, Aquaporins, Wallerian degeneration, Remak cells, Schwann cells, Nerve injury

## Abstract

In the peripheral nervous system aquaporins (AQPs) have been reported in both peripheral neurons and glial cells. Previously we described the precise localization of AQP1 in the rat sciatic nerve, which is present in both Remak and myelin Schwann cells, and is enriched in the Schmidt-Lanterman incisures. In this work, we found that AQP1 in mouse is only present in Remak cells, showing a different localization between these species. However, after nerve crush injury the level of AQP1 mRNA expression remains constant at all times studied in rat and mouse. We then performed RT-PCR of nine AQP (AQP1–9) isoforms from rat and mouse sciatic nerve, we found that in rat only five AQPs are present (AQP1, AQP4, AQP5, AQP7 and AQP9), whereas in mouse all AQPs except AQP8 are expressed. Then, we studied the expression by RT-PCR of AQPs in rat after nerve crush injury, showing that AQP1, AQP4 and AQP7 expression remain constant at all times studied, while AQP2, AQP5 and AQP9 are upregulated after injury. Therefore, these two closely related rodents show different AQP1 localization and have different AQPs expression patterns in the sciatic nerve, possibly due to a difference in the regulation of these AQPs. The expression of AQP1 in Remak cells supports the involvement of AQP1 in pain perception. Also, in rat the upregulation of AQP2, AQP5 and AQP7 after nerve injury suggests a possible role for these AQPs in promoting regeneration following injury.

## 1 Introduction

In the peripheral nervous system (PNS) there are a number of distinct glial cells. In peripheral nerve trunks, axons are ensheathed by non-myelinating or myelinating Schwann cells. Remak fibers grouped unmyelinated small-caliber C nociceptor fiber axons, which are enfolded by non-myelin Schwann cells or Remak cells processes. Each axon is sunk into an individual mesoaxon and each Remak fiber bundle is surrounded by basal lamina (Griffin and Thompson, 2008). Remak fibers have been associated with pain perception mainly due to the activation of nociceptor receptors in the primary afferent fibers, stimulated by thermal, mechanical or chemical stimuli (Yam et al., 2018). Myelin Schwann cells have an intimate relationship with axons, establishing a 1:1 relationship forming an internode and promoting saltatory conduction. Myelinated axons are organized in a series of distinct subdomains, each internode exhibits a striking longitudinal (from node to node) and radially (from the axon to the basal lamina) polarity that is essential for nerve impulse propagation (Pereira et al., 2012; Salzer et al., 2008; Salzer, 2015). This domain organization can be divided in compact myelin and non-compact myelin regions of specialized junctions between the layer of the myelin lamellae, which correspond to the abaxonal and adaxonal membranes. Along the internode are cytoplasmic pockets spiraling within the myelin known as Schmidt-Lanterman incisures (SLI) that provide a conduit between the inner and outer cytoplasm compartments, although the role of SLI is unknown. The adaxonal membrane and the axolemma are further organized into a series of distinct domains at the nodes of Ranvier: the nodal, paranodal and juxtaparanodal regions. This domain organization is crucial for axon function and integrity and its disruption seems to be important in myelin disorders (Buttermore et al., 2013).

When a peripheral nerve is injured a series of events occur referred to as Wallerian degeneration, involving axonal degeneration and myelin sheath degradation that starts mechanically with fragmentation into small ovoid-like structures, becoming unmyelinated repair Schwann cells (Jessen and Mirsky, 2019). These repair cells proliferate and form longitudinal cell strands, termed Bands of Büngner, that guide axon regrowth, ensuring remyelination of axons, reinnervation of their targets and, ultimately, regeneration of the nerve (Ramón y Cajal, 1928; Chen et al., 2007; Jessen et al., 2015; Jessen and Mirsky, 2019).

Aquaporins (AQPs) are members of a family of 13 small (21–34kD) integral membrane proteins in mammals, which form channels facilitating water transport and other small solutes bi-directionally across cell membranes in response to osmotic gradients (Agre et al., 2002). AQPs are present across all kingdoms of life and are involved in crucial metabolic processes. They are expressed and regulated in a tissue-dependent manner and have been shown to participate in many physiological and pathophysiological processes (Day et al., 2014; Madeira et al., 2016; Papadopoulos and Verkman, 2013). The majority of the AQPs are post-translationally regulated, either by translocation to the membrane, changing the number of AQP molecules present in the plasma membrane, or by conformational change or gating, altering water transport through individual AQPs. In both regulatory processes, phosphorylation of AQPs takes place (Nesverova and Thörnroth-Horsefield, 2019; Törnroth-Horsefield et al., 2010). AQPs may also be regulated by miRNAs (Gomes et al., 2018).

Most of the work on AQPs has been done in the central nervous system (CNS), but little in the PNS. In the PNS, AQP1, AQP2 and AQP4 have been reported to be in both peripheral neurons and glial cells, and have been shown to participate in both physiological and pathophysiological processes (Ma et al., 2012; Gazerani, 2017). These three AQPs have been involved in pain transmission associated with neurons of the trigeminal ganglion and dorsal root ganglia (DRG) that mediate nociception (Gazerani, 2017), but whereas AQP1 and AQP2 have been described in DRG small neurons, AQP4 is expressed in satellite glial cells of sensory neurons (Borsani et al., 2009; Kato et al., 2014; Oshio et al., 2006; Shields et al., 2007; Zhang and Verkman, 2015).

In mouse facial nerve AQP1 is localized in Schwann cells and in an injury facial model AQP1 is upregulated (Zhang et al., 2013). In sciatic nerve injury models, AQP1 increases in DRG small neurons and the dorsal horn. In AQP1 deficient mice DRG, axonal regeneration is impaired (Kaya et al., 2014; Zhang and Verkman, 2015). AQP2 expression also increases in small-diameter DRG neurons, but not in the spinal cord (Buffoli et al., 2009), while AQP4 is upregulated after injury in astrocytes of the spinal cord (Oklinski et al., 2015). In the sciatic nerve, AQP1 was first described briefly in mouse (Oshio et al., 2006; Zhang and Verkman, 2015) and human (Gao et al., 2006). However, we showed the precise localization of AQP1 within the rat sciatic nerve (Segura-Anaya et al., 2015). AQP1 is present in both Remak and myelin Schwann cells. In myelinating cells, AQP1 is expressed in non-compact myelin, enriched in the SLI and at the paradonal regions of the nodes of Ranvier (Segura-Anaya et al., 2015). Here, we show that AQP1 localization in the sciatic nerve is different in mouse and rat, and different AQP isoforms are expressed in mouse and rat. Also, changes in expression of AQP isoforms in rat sciatic nerve are observed after crush injury.

## 2 Experimental procedures

### 2.1 Teased nerve preparations

All procedures involving animals were carried out in accordance with the official Mexican norm for production, care and use of laboratory animals (NOM-062-ZOO-1999). Protocol (48447-Q) was approved by the Bioethics Committee of the School of Medicine of the Universidad Autónoma del Estado de México, minimizing the number of animals used and their suffering. Male Wistar rats (200–300 g) and CD1 mice (30–45 g) were used in all experiments. Animals were killed by CO_2_ overexposure. Crushed and normal sciatic nerves from rats and mice were excised and desheathed. The nerves were split into manageable strands and then teased gently onto poly-L-lysine-coated slides in a drop of PBS. Teased nerves were allowed to air-dry for at least 1 h before immunostaining.

### 2.2 Surgery

Rats and mice were anesthetized during surgery with ketamine (90 mg/Kg) and xylazine (10 mg/Kg). Anesthesia was confirmed and monitored by the absence of pain reflex. Surgery was performed as described (Lara-Ramírez et al., 2008). Briefly, the left leg muscles were separated at the mid-thigh level to uncover the sciatic nerve, which was then crushed. The sciatic nerves were crushed with fine forceps #7 (Dumont, Switzerland) for 30 s and animals were allowed to recover for 12 h, 1, 3, 5, 7, 9, 14, 21 and 30 days after crush. The undamaged contralateral nerves and normal sciatic nerves were used as controls.

### 2.3 RNA isolation

RNA was extracted from rat and mouse sciatic nerves, and also from brain, liver, and kidney, for use as controls. Total RNA was isolated by a single-step method using TRIzol^TM^Reagent (Thermo Fisher Scientific, Waltham, Massachusetts, USA) according to the manufacturer’s instructions with some modifications as previously reported (Segura-Anaya, 2015). Briefly, 50–100 mg of fresh tissue samples were homogenized in TRIzol (1 ml/100 mg tissue). The cleared homogenate was incubated for 5 min at 15–30 °C and then 0.2 ml of chloroform was added. After incubation for 3 min at 15–30 °C and centrifugation (12,000 x g for 15 min 4 °C) the aqueous phase was transferred to a fresh tube containing 0.5 ml of isopropanol and the precipitate collected by centrifugation (7500 x g for 5 min at 4 °C). The RNA pellet was incubated with 4 M LiCl for 15 min on ice and washed twice with 75% and 100% v/v ethanol, respectively. The pellet was briefly air dried, dissolved in ultrapure water, incubated for 10 min at room temperature and stored at − 80 °C until used. Total RNA from kidney, brain, and liver was extracted using the kit Quick-RNA MiniPrep (Zymo Research) according to the manufacturer’s instructions. The total RNA obtained was used for RT-PCR.

### 2.4 Primers

Both sense and antisense primers for AQP1, AQP2, AQP3, AQP4, AQP5, AQP6, AQP7, AQP8, and AQP9 from rat and mouse were used and for β-actin the same primers were used for both rodents. The primers were designed from the mRNA sequence of each AQP isoform using the Vector NTI advance program (Thermo Fisher Scientific, Waltham, Massachusetts, USA). The sequence of the primers (sense and antisense) for all the AQP isoforms and β-actin, and the expected sizes of the amplification products are shown in Table 1.

**Table 1:** Primer sequences used for RT-PCR amplification of AQP isoforms and β-actin.

Rat mRNA	**Forward primer (5′−3′)**	**Reverse primer (5′−3′)**	Product Size (bp)
**AQP1**	TCTGAGCATCGCTACTCTGG	AGAGCCACAGACAAGCCAA	**363**
**AQP2**	CTGGGCCACCTCCTTGGGATC	CCACCAGGGGTCCGATCCA	**124**
**AQP3**	CACTTGAACCCTGCTGTGACCT	GCTGCTGTGCCTATGAACTGATC	**269**
**AQP4**	TTGGACCAATCATAGGCGC	GGTCAATGTCGATCACATGC	**213**
**AQP5**	CTCTCACTGGGTCTTCTGGGTA	GGGTGCTTCAAACTCTTCGTCTTCC	**271**
**AQP6**	CTGCGGTCATTGTTGGGAAGT	CTGTGTCCTCTGAGTTCGTCTGTG	**219**
**AQP7**	CCTTGGTTCCGTGGCTCATAT	TTGTCCGTGATGGCGAAGATAC	**423**
**AQP8**	GAGCAGACGCCGATGTGTAGTAT	CCAGACGCATTCCAGAACCTT	**422**
**AQP9**	CCTGTTCTCTCGGACTCAACTCT	TTCTGCCTTCACTTCTGGGTCG	**230**
Mouse mRNA	**Forward primer (5′−3′)**	**Reverse primer (5′−3′)**	Product Size (bp)
**AQP1**	GACACCTGCTGGCGATTGACTAA	CCTCCTCTATTTGGGCTTCATCTC	**281**
**AQP2**	GCCCAGAGGAAGAGAGAAGAGA	GTGCCAATGCCCAGACCAAA	**255**
**AQP3**	CACTTGAACCCTGCTGTGACCT	GCTGCTGTGCCTATGAACTGATC	**269**
**AQP4**	TCTCCCATACTGCTTTGCCTTCC	GGTCCATTATCTCGCTGCCACA	**282**
**AQP5**	GCAACAACACAACACCAGGCAG	GTACCCAGAAGACCCAGTCAGA	**258**
**AQP6**	CTTAACGGCAAGAGCACAGGGA	GAGACCTTTTGGAAGAGCGACCT	**262**
**AQP7**	ATAAAGCACACCACCTATCAGCG	CAGAAAAGAGCCCAGAATCACT	**229**
**AQP8**	GAGCAGACGCCGATGTGTAGTAT	CCAGACGCATTCCAGAACCTT	**416**
**AQP9**	CCTGTTCTCTCGGACTCAACTCT	TTCTGCCTTCACTTCTGGGTCG	**230**
mRNA	**Forward primer (5′−3′)**	**Reverse primer (5′−3′)**	Product Size (bp)
**β-Actin**	AACACCCCAGCCATGTACG	ATGTCACGCACGATTTCCC	**254**

### 2.5 cDNA synthesis

Single-stranded cDNA was synthesized from 500 ng total RNA per sample. cDNA synthesis was carried out as previously described (Segura-Anaya et al., 2015).

### 2.6 Immunoblot analysis

Rat and mouse sciatic nerves and kidney tissue were homogenized directly in SDS solubilization buffer (2% w/v SDS, 2 mM EDTA, 2 mM EGTA, 5 mM Tris–Cl, 1 mM PMSF pH 7), and protease inhibitors cocktail (800 nM aprotinin, 40 μM bestatin, 100 μM leupeptin, 1 μg/ml pepstatin, 1 mM PMSF, Sigma-Aldrich). Total protein from sciatic nerves (25 μg), and rat (1 μg) or mouse (3 μg) kidneys were subjected to 10% SDS-PAGE and blotted as described (Segura-Anaya et al., 2015). The blot was incubated first with rabbit anti-AQP1 (1:3000; ProteinTech Group, Chicago, IL, USA) and then with biotinylated anti-rabbit antibody (1:3000; Vector, Burlingame, USA) and detected by chemiluminescence (ECL, Amersham).

### 2.7 Silver staining

We performed Bielschowsky’s silver staining method (BSSM) as previously reported (Segura-Anaya et al., 2018). Briefly, teased nerves were fixed in 4% w/v paraformaldehyde for 30 min and rinsed twice in PBS-A and distilled water. The slides were immersed in pre-warmed (37 °C) 10% w/v filtered silver nitrate, stained for 30 min and replaced with distilled water. To the silver nitrate solution in the flask, 6.0 ml of concentrated ammonium hydroxide were added, and the solution was cleared, which at this point is black, by adding, drop by drop, 1–3 ml of concentrated ammonium hydroxide until the precipitate that forms became clear. The slides were stained with this ammoniacal silver for 15 min on 37 °C, then exchanged for 1% w/v ammonium hydroxide solution for 3 min and returned to the ammoniacal silver added with a fresh developing solution for 3–5 min. The reaction was stopped, first with 1% w/v ammonium hydroxide for 3 min and then with 5% w/v sodium thiosulfate solution for 5 min. Nuclei were stained and slides mounted with Vectashield antifade mounting medium with DAPI (Vector, Burlingame, USA).

### 2.8 Simultaneous double-immunofluorescence and silver staining

Simultaneous double-immunofluorescence silver staining was performed as previously reported (Segura-Anaya et al., 2018). Briefly teased nerves were fixed in methanol for 15 min prior to incubation with rabbit anti-AQP1 (1:500, Proteintech cat. 20333–1-AP, Chicago, IL, USA) and stained with Alexa-fluor 488 (1:150; Jackson ImmunoResearch, West Grove, PA, USA). Then, the samples were double-labelled with mouse anti-GFAP conjugated with Cy3 (glial fibrillary acid protein) (1:1000, Sigma-Aldrich, St. Louis, MO, USA) or with mouse monoclonal anti-MBP (myelin basic protein) (1:1000; Calbiochem, San Diego, CA, USA) and stained with anti-mouse Alexa 568 (1:3000; Molecular Probes, Eugene, OR, USA). All antibody incubations were at 4 °C overnight. After that, Bielschowsky’s silver staining was performed as in Section 2.7, starting with re-fixing the samples with 4% w/v paraformaldehyde for 30 min. Controls were performed by omission of the primary antibody. Specimens were examined by epifluorescence on an Olympus BX60 microscope, using a QIClick^TM^QIimaging camera (Surrey, BC, Canada) followed by image processing with QCapture for Mac OS/X (Surrey, BC, Canada) and ImageJ 1.53a (National Institutes of Health, USA) for Mac OS/X.

### 2.9 Immunohistochemistry

To confirm the presence of SLI in mouse nerves we performed double-labelling with fluorescent phalloidin and anti-AQP1 as previously reported (Segura-Anaya et al., 2015). Briefly, teased nerves were fixed in 4% w/v paraformaldehyde for 20 min washed and incubated with phalloidin (2.5μl:100μl). The nerves were then refixed in methanol 10 min, washed and incubated with anti-AQP1 antibody (1:500) and stained with Alexa-fluor 488 (1:150).

### 2.10 Statistical analysis

Statistical analysis has been performed using RStudio software. Results are expressed as medians with interquartile ranges (25th; 75th percentile). The level of significance was set at P < 0.05. Differences between groups were examined for significance by performing one-way ANOVA with Tukey’s test. For the parametric test, distribution and homoscedasticity were tested using Shapiro-Wilk normality test and Levene’s test respectively. Data assumed variance homogeneity of AQP1, AQP2, AQP4 and AQP7. A square-root transformation was applied with AQP5 and AQP9 for getting a variance homogeneity.

## 3 Results

### 3.1 Expression of AQP1 in mouse and rat sciatic nerve

**Fig. 1:**
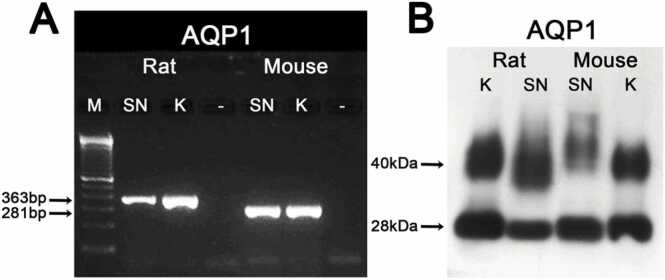
Expression of AQP1 in rat and mouse sciatic nerve. (A) RT-PCR analysis of AQP1 was performed using total RNA from the sciatic nerve (SN) and kidney (K) as control. AQP1 is expressed in both rat and mouse sciatic nerves. The fragments were analyzed on a 2% w/v agarose gel with a 100-bp molecular weight ladder (M). (B) Immunoblot analysis of AQP1 in the sciatic nerve and kidney. Anti-AQP1 recognizes a band of 28 kDa and a glycosylated band of 40–60 kDa in both rat and mouse.

We first determined the expression of AQP1 in the peripheral nerve by RT-PCR using total RNA from rat and mouse sciatic nerve, and kidney (as a control) using specific primers (Table 1). RT-PCR fragments show the presence of AQP1 in both species (Fig. 1A). We then performed immunoblot analysis with anti-AQP1 of the whole sciatic nerve and kidney (Fig. 1B). The results revealed two bands in both species one of ~ 25–28 kDa corresponding to the native form of AQP1, and a band of ~40–60 kDa which represents the N-glycosylated form of the former. In mouse, the glycosylated band shows a higher molecular weight than in rat. Thus, AQP1 mRNA and protein are present in both mouse and rat sciatic nerves.

### 3.2 Immunolocalization of AQP1 in rat and mouse sciatic nerve

We next determined the localization of AQP1 in rat and mouse sciatic nerves. AQP1 was examined by immunohistochemistry in preparations of teased nerves (Fig. 2.1). To identify Remak and myelin Schwann cells we double-labelled with mAbs against GFAP and MBP, respectively. The nerves were simultaneously stained by BSSM (Fig. 2.1A, D, G, J) and double-immunofluorescence against AQP1 and GFAP (Fig. 2.1B, H), or MBP (Fig. 2.1E, K). In rat, we have previously reported the localization of AQP1 in the sciatic nerve (Segura-Anaya et al., 2015), where AQP1 is strongly present in Remak cells, which co-distributed with GFAP (Fig. 2.1B) by immunofluorescence and silver staining (Fig. 2.1C). In myelinated Schwann cells, AQP1 is strongly localized in the SLI in the merged images of MBP immunofluorescence (Fig. 2.1E) and silver impregnation of axons (Fig. 2.1F). In mouse, AQP1 co-distributed with GFAP by immunofluorescence in Remak cells (Fig. 2.1H) and silver staining (Fig. 2.1I). However, in the merged images of myelin stained by MBP immunofluorescence (Fig. 2.1K) and silver impregnation of axons (Fig. 2.1L), AQP1 is not present in the SLI of myelinated Schwann cells. To corroborate whether the SLI are present in mouse, we double-labelled with AQP1 and phalloidin (as a marker for actin in the SLI), showing that mouse internodes have SLI, but do not express AQP1 (Fig. 2.1M-O). Thus, the results show that in rat AQP1 is present in both Remak and myelin Schwann cell, whereas in mouse AQP1 is present in Remak cells, but not in the SLI of myelinated internodes.

**Fig. 2:**
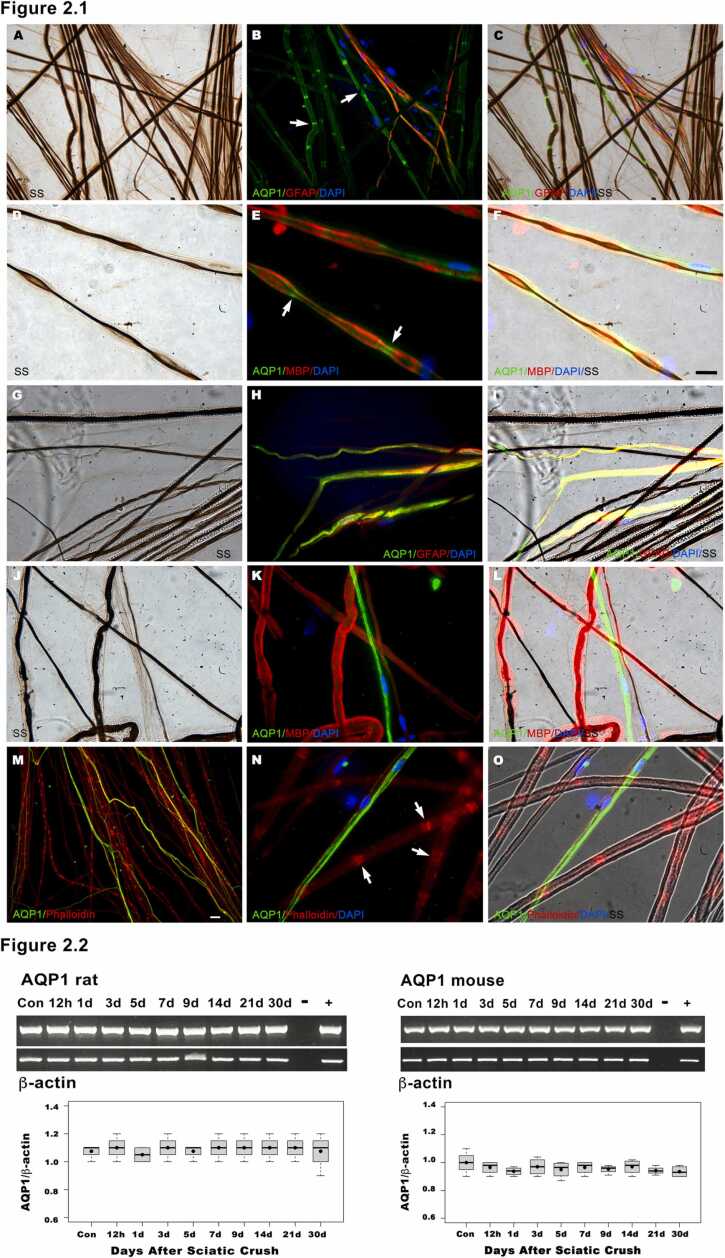
AQP1 is present in both myelin and Remak Schwann cells in rat, but is localized only in Remak Schwann cells in mouse (Fig. 2.1). Rat and mouse teased nerves were simultaneously stained with BSSM (A, D, G, J) and double-immunofluorescence with AQP1 and GFAP (B, H), or AQP1 and MBP (E, K) and merged (C, F, I, L) with the silver impregnation of axons (SS). In rat, AQP1 colocalizes with GFAP (B, C) in Remak cells and is present in the SLI (arrows in B, E) of myelin Schwann cells stained with MBP (E, F). In mouse, AQP1 colocalizes only with GFAP (H, I) in Remak cells, but not with MBP in myelin Schwann cells (K,L). Mouse teased nerves were double-labelled with AQP1 and phalloidin (M, N, O) showing the presence of SLI (arrows in N) stained with phalloidin in myelin Schwann cells, but not with AQP1, whereas in rat, AQP1 is strongly present in SLI (arrows in B, E). The cell nuclei were stained with DAPI (blue). Bar = 15 µm. After crush injury, the levels of AQP1 mRNA remain constant in both rodents (Fig. 2.2). Representative RT-PCR analysis of rat and mouse AQP1 was performed using total RNA from sciatic nerve (Con) as control and the distal region of the nerve at 12 h, 1, 3, 5, 7, 9, 14, 21, and 30 days after nerve crush. Relative expression values of each mRNA were normalized against β-Actin. The levels of AQP1 mRNA remain constant across all time points analyzed after crush injury in both rodents.

### 3.3 Expression of AQP1 in rat and mouse sciatic nerve after crush injury

We next asked whether there are also differences in rat and mouse AQP1 expression after nerve crush injury (Fig. 2.2). Rat and mouse sciatic nerves were crushed and the animals were allowed to recover for 12 h, 1, 3, 5, 7, 9, 14, 21 and 30 days after crush, and RT-PCR from the distal region of the nerves was performed. The results show that the expression of AQP1 remains constant at all times studied after crush injury in both rat and mouse sciatic nerves (Fig. 2.2).

### 3.4 Expression of AQP isoforms in rat and mouse peripheral nerve

**Fig. 3:**
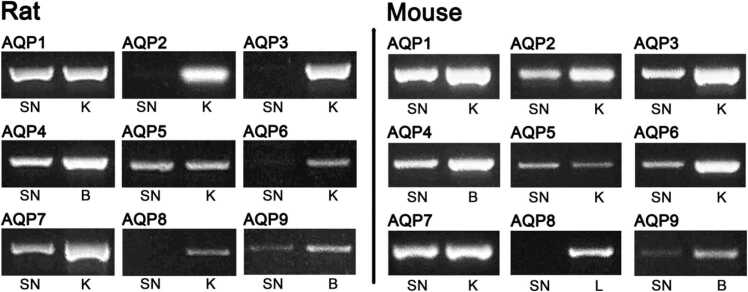
Different expression of AQPs between rat and mouse sciatic nerve. Representative RT-PCR analysis of AQP1, AQP2, AQP3, AQP4, AQP5, AQP6, AQP7, AQP8 and AQP9 using total RNA from rat and mouse sciatic nerve (SN) and as control tissues, kidney (K), brain (B), and liver (L). In rat, AQP2, AQP3, AQP6 and AQP8 are not expressed, while in mouse, only AQP8 is not present.

The difference in the localization of AQP1 between rat and mouse suggests the presence of other AQPs in the nerve. So, we then asked whether a difference in AQPs expression exists between rat and mouse sciatic nerves. We determined the expression of mammalian AQPs (AQP1-AQP9) by RT-PCR using total RNA isolated from rat and mouse sciatic nerves (Fig. 3). Reverse transcribed cDNA was PCR-amplified using specific primers (AQP1–9) (Table 1). There are five AQPs in rat and eight AQPs in mouse expressed in the sciatic nerve. In rat AQP1, AQP4 and AQP7 are strongly amplified, AQP5 is weakly amplified, while AQP9 is very weak. There are four AQPs not present in rat, AQP2, AQP3, AQP6 and AQP8 despite appropriate positive controls. AQP2 is very weakly present in some experiments, but most of the time is absent. We previously found that AQP1 was present in the nerve although we did not detect AQP2, AQP4 and AQP9 (Segura-Anaya et al., 2015). In this work, however, we find that increasing the sample concentration and designing different AQP4 and AQP9 primers we can detect the presence of AQP4 and AQP9 in the rat sciatic nerve. In mouse, all the AQPs are present, except AQP8. AQP1, AQP2, AQP3, AQP4 and AQP7 are expressed in higher quantities, while AQP5 and AQP6 are in lower quantities and AQP9 amplifies very weakly. Thus, the results show that there are differences in the expression of AQP isoforms between mouse and rat.

### 3.5 Expression of AQP isoforms in rat sciatic nerve after crush injury

**Fig. 4:**
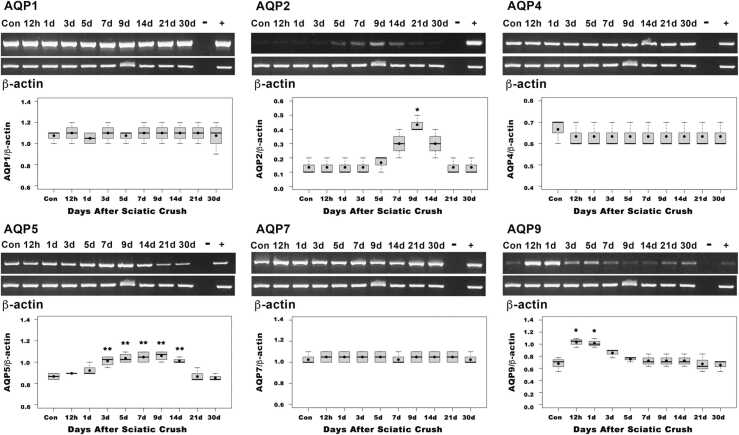
Expression of rat AQPs after crush injury. Representative RT-PCR analysis of rat AQP1, AQP2, AQP4, AQP5, AQP7 and AQP9 was performed using total RNA from the sciatic nerve (Con) as control, and the distal region of the nerve at 12 h, 1, 3, 5, 7, 9, 14, 21, and 30 days after crush nerve. Each box plot shows the data for AQP1, AQP2, AQP4, AQP5, AQP7 and AQP9 within the interquartile range, the median and mean are represented by a solid black and a black point respectively. Whiskers show maximum and minimum values (n = 3 or 4). After crush injury, the levels of mRNA of AQP1, AQP4 and AQP7 remain constant across all time points (p > 0.05), while AQP2 (p < 0.01,*), AQP5 (p < 0.001,**), and AQP9 (p < 0.01,*) change significantly at different time points analyzed. Relative expression values of each mRNA were normalized against β-Actin.

We next asked whether there are differences in rat AQPs expression after nerve injury. Rat sciatic nerve was crushed and the animals were allowed to recover for 12 h, 1, 3, 5, 7, 9, 14, 21 and 30 days after crush. RT-PCR was performed of the five AQPs present in the sciatic nerve and AQP2 (Fig. 4). We examined AQPs expression in the regenerating distal nerve stump following a sciatic nerve crush. We analyzed nine-time points after injury: 12 h and 1d, when fragmentation and degeneration of axons begins; 3d, when myelin fragmentation is well advanced; 5d and 7d, when repair SCs dedifferentiate and proliferate, macrophages infiltrate the nerve and myelin clearance occurs; 9d and 14d, when repair SCs organize in the bands of Büngner, axons grow and remyelination by SCs takes place; and finally, 21 and 30 days, when axons are reinnervating their targets and the nerve is regenerated (Fig. 4).

The results show that the expression of AQP1, AQP4, and AQP7 remains constant at all times studied after crush injury showing no statistically significant difference (p > 0.05) at any of the time points analyzed following injury versus normal sciatic nerve (control) (Fig. 4). In contrast, AQP2, is not present, or has very low levels of expression in normal sciatic nerve, but starts increasing at day 5, reaching a significant peak at day 9 after injury compared to non-injured nerves (p < 0.01) and returning to normal levels by day 30. AQP5 shows a significant increase at day 3 and remains like that until day 14 (p < 0.001), returning to its normal level by day 30. AQP9, which also has very low levels in the normal sciatic nerve, is significantly upregulated to double its normal level immediately at 12 and 24 h after injury (p < 0.01), and returns to its normal levels by day 7 (Fig. 4). Thus, the results show that in the rat AQP1, AQP4 and AQP7 mRNA expression remains constant after crush injury, while AQP2, AQP5 and AQP9 expression is upregulated after injury.

## 4 Discussion

Our results show that AQP1 is present in mouse and rat, but unexpectedly, its immuno-localization in the sciatic nerve is different. While in rat it is present in both myelin Schwann cells and Remak cells, in mouse AQP1 is only present in Remak cells. We have also performed immune-localization in guinea pig sciatic nerve and found that AQP1 is localized only in Remak cells as it is in mouse (data not shown). It would be interesting to know the localization of AQP1 in human sciatic nerve. Very recently, single cell sequencing data of mouse peripheral nerves have been used to build a sciatic nerve atlas from early development through to the adult stage (Gerber et al., 2021). They performed single-cell RNA sequencing analysis of Schwann cells at different stages of postnatal differentiation. This allows one to follow the trajectory of myelinating and non-myelinating Schwann cells at different stages of development through to the adult stage. Using this atlas for AQP1 (https://www.snat.ethz.ch/), we observe that the expression of AQP1 in mouse in the single-cell RNA sequencing data at postnatal day 60 (P60) shows that AQP1 is only present in non-myelinated Schwann cells or Remak cells. Therefore, these results are in agreement with ours, showing the localization of AQP1 only in Remak cells by immunohistochemistry of teased mouse sciatic nerves. Differences in the localization of AQP1 has been described in the trigeminal systems between human and mouse (Gao et al., 2012). AQP1 is expressed in small and medium-sized trigeminal neurons in both human and mouse, but while all human satellite cells express AQP1, in mouse satellite cells AQP1 is only localized in the subset of trigeminal neurons, appropriately termed AQP1-positive. Also, in the trigeminal roots of humans AQP1 is present in astrocytes, while in mice it is localized in neurons (Gao et al., 2012). AQP4 polarization in astrocytes is different between mouse and human (Eidsvaag et al., 2017). In mouse, it is prominently clustered around vessels facing the capillary endothelium while in human it is less polarized and has a higher AQP4 expression in parenchymal astrocytic membranes (Eidsvaag et al., 2017). There is a possibility that the difference in AQP1 localization between rat and mouse is due to changes in the protein structure. Therefore, we compared the amino acid sequence of AQP1 between mouse and rat with a popular sequence alignment algorithm, such as BLAST, finding that the sequences have 98% of identity showing a high degree of homology. This suggests that the different AQP1 localization in the nerve is not at the protein structure, but at a possible difference in the regulation of AQP1 in the nerve between both rodents. Also, the fact that AQP1 is localized in Remak fibers, suggests that it is involved with pain perception due to the activation of nociceptor receptors in the primary afferent fibers. AQP1 has been observed in a subpopulation of small neurons of DRG and trigeminal ganglion (Oshio et al., 2006; Shields et al., 2007; Zhang and Verkman, 2015). A role of AQP1 in pain signal transduction (Oshio et al., 2006) and a reduced behavioral response to inflammatory, thermal and cold induced pain has been reported using AQP1 knockout mice (Zhang and Verkman, 2010). However, Shields et al., 2007 showed that AQP1 is not required for normal pain processing, neither electrophysiological nor behavioral tests uncovered differences in nociceptive processing between mice lacking AQP1 and wildtype mice. This controversy concerning the role of AQP1 in the perception of pain remains to be resolved. However, the localization of AQP1 in Remak cells of the rodent sciatic nerves we studied and in small DRG neurons that give rise to unmyelinated C-fibers (Shields et al., 2007) suggests a functional role of AQP1 in nociceptive processing.

We then showed that the expression of mammalian AQPs (AQP1-AQP9) is different in rat and mouse sciatic nerve. There are five AQPs present in rat (AQP1, AQP4, AQP5, AQP7 and AQP9), while in mouse all AQPs except AQP8 are expressed. We consulted the sciatic nerve atlas (https://www.snat.ethz.ch/) for the AQPs we studied and we observe that in the atlas, all the AQPs are expressed in mouse sciatic nerve except AQP6 and AQP8. The absence of AQP8 is in agreement with our results, but we observed expression of AQP6 in the nerve, one possibility is that AQP6 is not present at the protein level. A similar study to our work by RT-PCR in mouse spinal cord, showed that only AQP4, AQP5, AQP8, and AQP9 are expressed, while AQP1, AQP2, AQP3, AQP6 and AQP7 are absent. However, AQP5 was not detected by immunohistochemistry and AQP8 is only localized in ependymal cells lining the central canal of the spinal cord (Oshio et al., 2004). Also, it has been shown that AQP1 protein originates in the DRG and expression of AQP1 mRNA is not expected within the spinal cord (Nesic et al., 2010). Another work in heart showed, by RT-PCR, different AQP isoforms-expression between mouse, rat and human (Butler et al., 2006). These results and ours suggest that different tissues express a different set of AQP isoforms, and we might speculate that similar functions in several tissues may involve the participation of different AQP isoforms, which may lead to multiple pathways considering the possible differences in the regulation of AQPs among species.

Evolutionary analysis of the AQPs family showed paralogue groups, or subdivisions, based on molecular evolution by gene duplication followed by functional and structural specialization (Heymann and Engel, 1999; Zardoya and Villalba, 2001). Mammalian AQPs (AQP0–9) are classified into three groups: the AQPs specific for water AQP0, AQP1, AQP2, AQP4, AQP5, and AQP6; the AQPs that also transport glycerol and urea AQP3, AQP7 and AQP9; and the metazoan AQP8 (Heymann and Engel, 1999; Zardoya and Villalba, 2001). The analysis shows that AQP8 has a separate phylogenetic origin from the other AQPs, and has an unusual primary structure characterized by a long amino-terminal and a short carboxyl end (Koyama et al., 1998). In the CNS, AQP8 has been reported in oligodendrocytes, neurons and astrocytes in culture, and in ependymal cells lining the central canal of the spinal cord (Xu et al., 2017). Therefore, our results show that AQP8 is not expressed in both rodents and that only the AQPs of the same phylogenetic origin are present in these nerves.

We next looked for changes in the level of expression of rat AQPs during crushed injury. Our results show that the levels of mRNA expression of AQP1 (in both rat and mouse), AQP4 and AQP7 remain constant during Wallerian degeneration, while a significant upregulation of AQP2, AQP5 and AQP9 expression is observed after injury. In this study, AQP2 mRNA was absent in most of the normal rat sciatic nerves, although it was detectable in a few rat sciatic nerve samples, but only at very low levels. This is in agreement with other studies that have not observed AQP2 in rat and mouse normal sciatic nerve (Borsani et al., 2009; Buffoli et al., 2009; Albertini and Bianchi, 2010). However, in a mouse inflammatory pain model, AQP2 is present in Schwann and satellite cells of the trigeminal ganglion (Borsani et al., 2009). Also, after chronic constriction of the rat sciatic nerve, AQP2 is localized in small neurons and Schwann cells of the DRG (Buffoli et al., 2009; Albertini and Bianchi, 2010). Therefore, we decided to study the expression of AQP2 in the nerve after crush injury together with the other AQPs expressed in the rat. We found that AQP2 is upregulated after day 5 reaching a peak at day 9 after injury, during the time in which the bands of Büngner are formed and the axons regenerate through it; while AQP5 increases after day 3 and remains like that until day 14 after injury, the time in which all the processes of regeneration of axons and remyelination takes place. In contrast, upregulation of AQP9 was observed much earlier at 12 and 24 h after injury, and soon after returned to normal, the time in which triggers the process of axons degeneration and formation of myelin ovoids-like structures (Ramón y Cajal, 1928). Thus, these results show that each of these AQPs are involved at different stages of Wallerian degeneration and might, therefore, have a different role in these events. It would be interesting to know the detailed localization and role of these AQPs during this process.

It is possible that AQPs that are expressed at higher levels (AQP1, AQP4 and AQP7) in the sciatic nerve, but remain constant after injury, are regulated not through mRNA transcription, but post-transcriptionally by miRNAs. Several human pathologies have been associated with AQPs miRNA deregulation (Gomes et al., 2018). In rat cerebral ischemia miR-320a inhibits AQP1 and AQP4 expression, whereas anti-miR-320a upregulates AQP1 and AQP4 expression, identifying miR-320a as a potential modulator of these AQPs (Sepramaniam et al., 2010). Therefore, there is a possibility that the levels of AQP1, AQP4 and AQP7 mRNA expression do not change, but are regulated by miRNAs after nerve injury. Another possibility is due to changes in AQP sub-cellular localization. It has been proposed that a regulatory mechanism of AQP isoforms is a calcium or phosphorylation-dependent event, including translocation to and from the plasma membrane, and, in the case of gating, whether the AQP is open or closed (Nesverova and Thörnroth-Horsefield, 2019; Conner et al., 2013). It has been reported that the AQPs (AQP0–12) identified in mammals are expressed and regulated in a tissue-dependent manner (Nesverova and Thörnroth-Horsefield, 2019). In this report, the results show from the nine AQPs studied (AQP1–9) only five AQPs are expressed in rat, whereas eight AQPs are present in mouse. Also, the localization of AQP1 is different between mouse and rat. Thus, the results suggest that the regulation of AQPs differs among species and might differ with respect to tissues.

Rodents have been widely used as models in biomedical research: rats (Rattus norvegicus) and mice (Mus musculus), belonging to the order Rodentia. However, the fact that AQP1 expression is different in mouse compared to rat must be taken into consideration when translating physiological and pathophysiological findings into humans in a clinical setting.

## 5 Conclusion

The localization of AQP1 and expression patterns of AQP isoforms in rat and mouse sciatic nerve are different and in rat AQP2, AQP5 and AQP9 are upregulated after nerve crush injury.

## Ethical statement

All procedures related to animal maintenance and experimentation were approved by the Committee for Animal Experiments at the Universidad Autónoma del Estado de México. The work described involved the use of sciatic nerves from laboratory rats that were kept and handled according to the official Mexican norm for production, care and use of laboratory animals [NOM-062-ZOO-1999].

## Funding sources

This work was sponsored by public funds through the research grant number UAEM 6256/2020CIB from the Universidad Autónoma del Estado de México (UAEM), México, awarded to the corresponding author.

## CRediT authorship contribution statement

All authors had full access to this study’s data and take full responsibility for its integrity and the accuracy of the data analysis. Contribution: conceived and designed the experiments: **MAR Dent.** Performed the experiments: **E. Segura-Anaya, A. Martínez-Gómez**. Analyzed the data: **MAR Dent, E. Segura-Anaya, A. Martínez-Gómez.** Figures preparation: **A Martínez-Gómez, E. Segura-Anaya.** Wrote the paper: **MAR Dent.**

## Conflicts of interest

None
